# Farber Disease Mimicking Juvenile Idiopathic Arthritis: The First Reported Case in Qatar and Review of the Literature

**DOI:** 10.1155/2022/2555235

**Published:** 2022-02-10

**Authors:** Amal Al-Naimi, Haneen Toma, Sara G. Hamad, Tawfeg Ben Omran

**Affiliations:** ^1^Pediatric Pulmonology Department, Sidra Medicine, Doha, Qatar; ^2^Department of Medical Genetics, Hamad Medical Corporation, Doha, Qatar; ^3^Division of Genetic and Genomic Medicine, Sidra Medicine, Doha, Qatar; ^4^Weill Cornell Medical College, Doha, Qatar

## Abstract

Farber disease (FD) is an extremely rare autosomal recessive disorder caused by the deficiency of lysosomal acid ceramidase. It is characterized by a triad of progressive multiple joints' involvement, subcutaneous nodules, and hoarseness of voice. In this report, we describe a 23-month-old boy diagnosed with Farber disease. Initially, he was misdiagnosed as juvenile idiopathic arthritis (JIA) because he presented with joint swelling. However, the associated hoarseness of voice, subcutaneous nodules, and poor response to treatment all have questioned the diagnosis of JIA and prompted the suspicion of Farber disease as an alternative diagnosis. The diagnosis was later confirmed genetically by the presence of a homozygous pathogenic variant (p.Gly213Glu; c.638G > A in exon 8) in the ASAH1 gene. The present case illustrates the diagnostic journey of a child with Farber disease as well as highlights that FD should be considered in the differential diagnosis of early onset arthritis in the presence of subcutaneous nodules and/or hoarseness of voice.

## 1. Introduction

Farber disease (FD) is an extremely rare, inherited, progressive lysosomal storage disease that is characterized by subcutaneous nodules, arthralgia, and hoarseness of voice. Due to its rarity, it can be often misdiagnosed as juvenile idiopathic arthritis (JIA).

Hereby, we present a case of Farber disease in a child who was initially managed as JIA. Our case represents the first reported case in the state of Qatar and highlights the importance of considering such rare genetic disease entities, especially in the context of lack of improvement with conventional therapy.

## 2. Case Presentation

**Figure 1:**
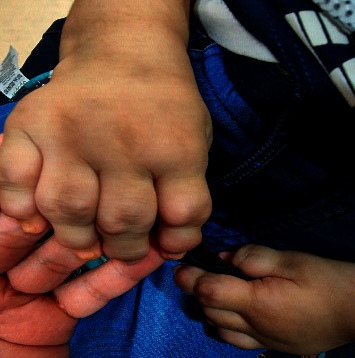
A picture of the patient's hands showing the swelling and deformities of the interphalangeal joints.

**Figure 2:**
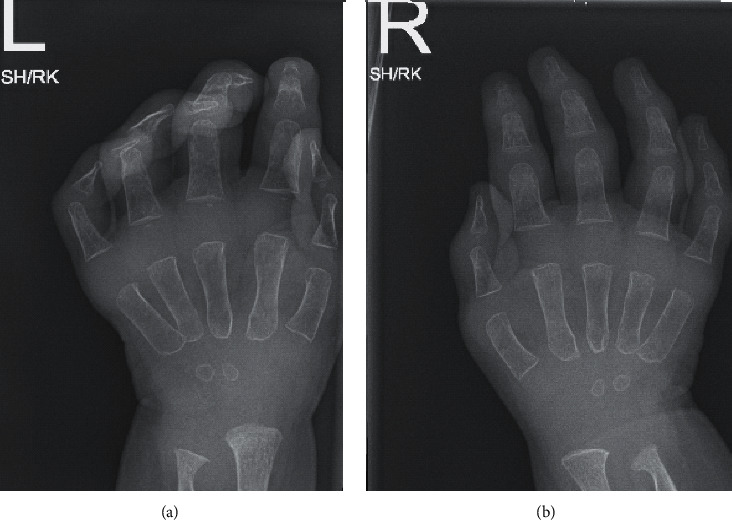
X-ray of the patient's hands (left: labelled L and right: labelled R) showing soft tissue swelling of both distal hands with grossly maintained joint spaces.

The patient is a 23-month-old boy with uneventful perinatal and postnatal history. He was referred to the pulmonology clinic with hoarseness of voice for the assessment of upper airway obstruction. His past medical history started at the age of 4 months with progressive hoarseness of voice and stridor. These symptoms were followed by swollen painful joints, predominantly in the hands and feet, in addition to poor weight gain. He was born to consanguineous parents of Indian descent with no family history of rheumatological or inherited disorders. Family sought medical advice at the age of 12 months. During the initial assessment in the pediatric clinic, the patient was found to have failure to thrive. His vital signs were stable, and he maintained oxygen saturation on room air. He had biphasic stridor with hoarse cry and intermittent subcostal retractions. The interphalangeal joints, wrists, knees, and ankles were swollen and tender with subcutaneous nodules on the wrist and ankles, as shown in Figure 1. There was no erythema or warmth. Bilateral hands' X-ray revealed soft tissue swelling of both distal hands with grossly maintained joint spaces. However, irregular borders of the distal metaphysis of metacarpals with scalloping of the distal ulna (Figure 2) were observed. Ultrasound of affected joints of the hands and feet revealed bulky hyperemic synovium and multiple foci of tenosynovitis without joint effusion. The working diagnosis at that point was juvenile idiopathic arthritis, so the patient was referred to Rheumatology service where he was commenced on multiple medications that were upgraded sequentially due to the lack of improvement, including systemic steroids and immunosuppressant medications. The biopsy that was taken from the subcutaneous nodules showed fibrosis, foamy macrophages, and chronic inflammatory cells without central necrosis.

**Figure 3:**
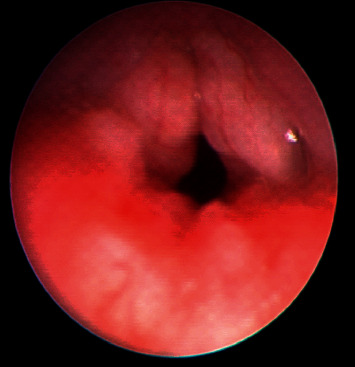
Bronchoscopic view of the supraglottic area showing the laryngeal nodular formation and narrowing.

At 20 months of age, he was referred to ENT clinic for persistent hoarseness of voice and stridor. Direct laryngobronchoscopy was done and showed multiple nodules on both vocal cords with significant narrowing and edema of the larynx, as shown in Figure 3. He was later referred to our chest clinic for further evaluation. Based on the clinical presentation and lack of response to the given therapy, Farber disease was highly suspected and was later confirmed genetically by genetic testing that revealed a homozygous mutation in the ASAH1 gene (p.Gly213Glu (GGA > GAA): c.638G > A in exon 8). Screening for other systemic involvement was unremarkable as evident by unremarkable chest CT and brain MRI.

During preparation for bone marrow transplant, the patient developed acute viral lower respiratory tract infection that worsened his upper airway obstruction and required respiratory support with noninvasive ventilation. Due to failure of weaning from noninvasive ventilation, the decision of tracheostomy tube insertion was taken, and the patient did not require any supplementary ventilatory support through the tracheostomy tube.

## 3. Discussion

Farber disease, also known as ASAH1-related disorders, is an autosomal recessive lysosomal storage disease. It is caused by mutations within ASAH1 encoding for acid ceramidase leading to its deficiency [1, 2]. The acid ceramidase deficiency results in altered apoptosis process and accumulation of ceramide in several cell types such as histiocytes and epithelial cells in multiple organs, including the skin, brain, liver, kidney, and lungs, leading to granulomatous formation [3]. Farber disease is extremely rare with an estimated prevalence of ≤1/1,000,000. The majority of patients with Farber disease were reported from India and the United States [4].

Farber disease classically presents with the triad of subcutaneous nodules, arthralgia, and hoarseness of voice [5]. The disease has seven subtypes depending on the age of onset and systematic involvement with variable severity and life expectancy. Our patient was classified as Farber disease-type 1 which is the classic and most common form of Farber disease. It usually presents at around 3–6 months of age as in our patient with early appearance of subcutaneous nodules, joint involvement, and hoarseness of voice. The joint involvement in type 1 is characterized by progressive polyarticular pain swelling and stiffness leading to significant joint deformities, especially of the hands and feet, whereas hoarseness of voice is caused by the progressive laryngeal nodular formation, which may lead to life-threatening upper airway obstruction and respiratory insufficiency mandating securing airway patency by the tracheostomy tube as occurred in our patient. Type 1 Farber disease may progress to affect the heart, central nervous system, and lungs with life expectancy of around 3 years [6, 7]. Patients with type 2 and type 3 usually have a longer life expectancy due to lesser neurological predilection. On the contrary, patients with type 5 have progressive neurological dysfunction which begins at 12 to 30 months of life. Type 4 is the most severe form which usually presents in neonates with hydrops fetalis, severe hepatosplenomegaly, and rapid neurological deterioration leading to death within the first few weeks of life [8]. Type 6 is a combination of Farber disease and Sandhoff disease which is an inherited neurodegenerative disorder [9]. Finally, the least common type is type 7 that is caused by a mutation of prosaposin leading to a combined deficiency of glucocerebrosidase, galactocerebrosidase, and ceramidase [10]. The more recent publications tend to simplify the classification of Farber disease into the classic childhood or the mild and attenuated form [11–13].

Due to its rarity, Farber disease is often underrecognized or misdiagnosed. In a recent retrospective study of 22 patients, Solyom et al. [14] reported that around 71% of the mild or moderate cases might be initially managed as JIA due to similarities of age of onset and joint involvement [15]. Severe neonatal form can also be misdiagnosed as reported in a neonate who was initially diagnosed with neonatal hepatitis and underwent liver transplant [8, 16].

The diagnosis is established by genetic testing which would show a homozygous mutation in the ASAH1 gene. Prenatal diagnosis might be performed in familial cases of Farber disease by examining the amniotic fluids by DNA testing or measurement of acid ceramidase activity in cultured amniotic fluid cells or chorionic villi [17–21].

Currently, there is no definitive treatment for Farber disease. Management is supportive and is guided towards alleviation of symptoms and alleviation of complications. Anti-inflammatory medications and physical therapy are mainly used to control pain and to improve the mobility of the joints [13].

Previous articles studied the benefit of hematopoietic stem cell transplantation (HSCT) in Farber disease and showed improvement only in joints' manifestation with no effect on the progression of the neurological involvement. Therefore, HSCT is recommended in patients with FD after exclusion of neurological involvement [22–24]. Our patient was considered a candidate for HSCT as he did not have any neurological involvement at the time of assessment.

Other therapeutic options such as gene and enzyme replacement therapies [25] are still experimental and evolving.

## 4. Conclusion

Our case represents an extremely rare lysosomal disorder that can be easily misdiagnosed as JIA. It highlights the importance of considering the diagnosis of rare genetic disorders, especially in the refractory cases despite optimal treatment and the presence of consanguinity. Early recognition of such diseases would aid in early management and monitoring of associated complications. Moreover, it provides a unique opportunity to highlight that reaching the final genetic diagnosis would also help in providing appropriate genetic counseling including future reproductive plans.
